# Effects of three contraceptive methods on depression and sexual function: An ancillary study of the ECHO randomized trial

**DOI:** 10.1002/ijgo.13594

**Published:** 2021-03-02

**Authors:** Mandisa Singata‐Madliki, Florence Carayon‐Lefebvre d’Hellencourt, Theresa A. Lawrie, Yusentha Balakrishna, G. Justus Hofmeyr

**Affiliations:** ^1^ Effective Care Research Unit Eastern Cape Department of Health/Universities of the Witwatersrand and Fort Hare East London South Africa; ^2^ FHI 360 Durham NC USA; ^3^ Evidence‐based Medicine Consultancy Ltd Bath UK; ^4^ Biostatistics Research Unit South African Medical Research Council Durban South Africa; ^5^ Department of Obstetrics and Gynecology University of Botswana Gaborone Botswana

**Keywords:** contraception, depression, depot medroxyprogesterone acetate, implant, intrauterine device, libido, sexual function

## Abstract

**Objective:**

To compare the effects of depot medroxyprogesterone acetate (DMPA‐IM), levonorgestrel (LNG) implant, and copper intrauterine device (IUD) on mood and sexual function.

**Methods:**

At the Effective Care Research Unit in South Africa, women already randomized in the ECHO Trial to the three methods were asked to participate in this study. Participants were interviewed at 3 and 12 months after enrollment using the Beck Depression Inventory and Arizona Sexual Experiences Scale, and at 12 months using the WHO‐5 Wellbeing Index and the Patient Global Impression scale.

**Results:**

A total of 605 women participated. There was little difference in depression at 3 months across the three study groups. Contrary to our hypothesis, at 12 months, depression was lowest among DMPA‐IM users (16/167, 9.6%) and highest among IUD users (28/158, 17.7%) (*p *= 0.032). There was little difference in sexual function at any time‐point. More women in the DMPA‐IM group felt “very much better” on the PGI scale than in the IUD and LNG implant groups (*p *= 0.003).

**Conclusion:**

Depression may be less likely with DMPA‐IM than with the other methods 1 year after initiation. Major differences in sexual functioning are unlikely. Unhappiness related to not using DMPA‐IM, the most popular method in our setting, may have skewed results.

Trial registration number: PACTR201706001651380.

## Synopsis

Depression at 12 months after initiation may be less likely with DMPA‐IM than the IUD and LNG implant; major differences in sexual functioning are unlikely.

## 1 INTRODUCTION

Contraceptive discontinuation is a major issue that impacts unintended pregnancy rates.^1^ Therefore, understanding the reasons for discontinuation is necessary to develop effective contraceptive services with appropriate counseling to inform women's contraceptive choices, and to manage adverse effects appropriately should these arise. Discontinuation rates for injectable progestins and the copper intrauterine device (IUD) in our setting are high, reported in a large locally conducted randomized trial as 16.5% and 14.7%, respectively, at a median of 20 months after initiation.^2^ Furthermore, in the recently published Evidence for Contraceptive options and HIV Outcomes (ECHO) Trial in which our Effective Care Research Unit participated, contraceptive discontinuation was associated with 71% of 181 pregnancies that occurred during the course of the trial.^3^

Despite their common occurrence, differences in adverse effects between contraceptive agents remain poorly elucidated. Specifically, the effect of contraception on mood has been under‐studied, particularly in low‐ and middle‐income countries, with published reports from high‐income countries suggesting the possibility of both an increase and a decrease in depression risk.^4^, ^5^, ^6^, ^7^ A nationwide cohort study from Denmark found that, compared with non‐users, use of various types of hormonal contraception was associated with a higher risk of subsequent depression and use of antidepressants ^4^; hormonal contraception has also been linked with suicide attempts and suicide.^5^ In contrast, a national study from the USA reported reduced levels of depressive symptoms among sexually active women taking hormonal contraception.^6^ In addition, a Swedish cohort study found an association between contraception and the use of psychotropic medicine among adolescents.^7^ The effects of contraceptives on mood may be complex, including, for example, biological effects on endogenous steroid hormones and psychological effects, such as relief from anxiety about unintended conception. The only study to date that has evaluated biological and psychological effects related to contraception was a randomized controlled trial (RCT) conducted in South Africa, which found increased postnatal depression among women using progestin‐only contraception.^8^ Such confusing and contradictory findings suggest that robust evidence from randomized trials is needed, but such studies are rare because of anticipated difficulties in randomizing women to different contraceptive agents.

To our knowledge, no RCTs have evaluated the relationship between contraceptive use and depression, if any, among women outside of the postnatal period. Limited evidence from two small RCTs conducted among postnatal women in South Africa have shown that, when administered soon after pregnancy, the risk of postnatal depression may be increased with injectable progestin contraception.^8^, ^9^ However, as a result of the short follow up of women in these trials, it is not known whether this adverse effect of progestin‐only contraception resolves or remains with longer‐term use. Similarly, the impact of contraceptive agents on sexual function has not been well‐studied and limited evidence suggests that they may decrease or increase libido, or have no effect.^10^

The multicenter ECHO Trial randomized women to intramuscular depot medroxyprogesterone acetate (DMPA‐IM), the IUD, and the levonorgestrel (LNG) implant and evaluated the effect of these agents on HIV incidence. The trial, therefore, provided a unique opportunity for us to also compare effects on depression and sexual function within the context of a stringent RCT protocol.^3^ We hypothesized that the progestin‐only contraceptives DMPA‐IM and the LNG implant may be associated with lower mood and more sexual dysfunction compared with the IUD.

## 2 MATERIALS AND METHODS

### 2.1 Design and participants

The protocol for this study was published in *BMJ Open* in 2018.^11^ The study is ancillary to the ECHO trial, which was a large multicenter RCT with three parallel arms (1:1:1). In the ECHO trial, participants were randomized to three contraceptive methods (IUD, DMPA‐IM, and LNG implant) and the effects on HIV incidence and pregnancy rates were compared (ECHO trial registration: NCT02550067.^3^ The trial was conducted at sites in Kenya, South Africa, Swaziland, and Zambia. Participants were sexually active, HIV seronegative women between the ages of 16 and 35 years who were seeking effective, long‐acting contraception and who gave informed consent. Exclusion criteria included using any of the study contraceptive agents in the preceding 6 months and planning a pregnancy in the next 18 months.

In the ECHO trial, 7829 women in total were randomized and followed up for 18 months. The findings, which were published in the *Lancet* in 2019, showed no clear differences in HIV incidence and pregnancy rates between the three contraceptive methods evaluated.^3^ No data on mental health were collected in the main trial.

The objectives of this ancillary study were to compare the effects of DMPA‐IM, the IUD, and the LNG implant on mood and sexual function. Participants were drawn from one of the trial centers, the Effective Care Research Unit, in East London, South Africa, and additional informed consent was obtained. Out of 7829 women enrolled in the ECHO trial, 615 came from our center; of these, 605 consented to participate in the ancillary study.

### 2.2 Procedure

Following online registration, the ECHO trial centrally randomized participating women to DMPA‐IM (150 mg/ml, Depot‐Provera^®^; Pfizer, Puurs, Belgium) given intramuscularly every 12 weeks, insertion of the Copper IUD (Optima TCu380A^®^; Injeflex, Sao Paolo, Brazil) at enrollment, or insertion of the subdermal LNG implant (150 mg, Jadelle^®^; Bayer, Turku, Finland) at enrollment. Participant and personnel blinding was not possible because of the nature of the interventions, and participant follow up was conducted at 1, 3, 6, 9, 12, 15, and 18 months. ECHO participants at our site who additionally consented to participate in this ancillary study were enrolled at the 1‐month ECHO follow‐up visit, and were interviewed at the 3‐ and 12‐month visits for this purpose by trained research assistants. The 12‐month follow up was an extension of the protocol that had originally planned a 3‐month follow up only. Some participants (*n* = 53) only contributed data at the 12‐month visit.

### 2.3 Outcomes and instruments

Primary outcomes of this ancillary study were depression and sexual dysfunction. Depression was evaluated using the Beck Depression Inventory (BDI‐II) and sexual function was evaluated using the Arizona Sexual Experiences Scale (ASEX).^12^, ^13^ The BDI‐II has previously been translated into the local language (IsiXhosa), validated, and used in our study population.^14^ The ASEX scale is a five‐item scale that quantifies sex drive, arousal, vaginal lubrication, ability to reach orgasm, and satisfaction from orgasm.^14^ We have used the ASEX scale in a previous study in our population and found it to be a useful tool although, to our knowledge, it has not been formally validated in this context.^9^ Following enrollment into the ancillary study at the 1‐month ECHO trial visit, these scales were administered by trained research assistants at the 3‐month and 12‐month assessments.

As mood and an individual's perception of well‐being are related, we also asked participants to complete the World Health Organization (WHO) Well‐being Index, which consists of five statements on well‐being that can be rated from 0 to 5.^15^ The well‐being score, which can range from 0 to 25 with 0 representing worst possible and 25 representing best possible quality of life, was calculated by adding the figures of the five answers. In addition to the WHO Well‐being Index, we used the Patient Global Impression Scale (PGI). This consists of one question on an individual's perception of the effect of a treatment on their well‐being.^16^ The WHO Well‐being Index and PGI scale were administered at the 12‐month follow‐up visit. Each of these tools can be self‐administered, but to optimize the quality of the data, trained research assistants asked and explained the instrument questions to participants in their local language, and recorded participants’ responses.

### 2.4 Statistical analysis

The ECHO trial was designed to detect a 50% difference in HIV incidence between any of the three contraceptive methods with 80% power.^3^ For the ancillary study, we assumed a reduction in the incidence of depression with the alternative contraceptive methods compared with DMPA‐IM of 50% based on a rate of depression with DMPA‐IM of 26.5% from a previous study conducted in our setting.^9^ We calculated that we would need at least 158 participants in each arm to detect a difference with 80% power between any of the three arms.^11^ Stata Statistical Software V.15 (StataCorp., College Station, TX, USA) ^17^ was used for analysis, which was performed by YB. We used standard BDI‐II thresholds to categorize any depression (≥14), moderate depression (≥20) and severe depression (≥29)^12^; and the standard ASEX threshold to categorize sexual dysfunction (≥19) among other suggested criteria.^13^ As per the preplanned analysis, we performed separate pairwise χ^2^ tests between each of the three methods. The χ^2^ test was used to compare categorical outcomes, unless events were equal to or less than five; in these instances, the Fisher's exact test was used. As per the pre‐planned analysis, we performed separate pairwise χ^2^ (or Fisher's exact) tests between each of the three methods. Mean scores were compared using the Kruskal‐Wallis test and for non‐normally distributed data we used the Wilcoxon test to compare medians and interquartile ranges. Analysis was by intention‐to‐treat.

### 2.5 Ethical approval and study registration

All participants gave written informed consent to separately participate in this ancillary study, which received ethical approval from the University of the Witwatersrand Committee for Research on Human Subjects on February 17, 2016 (Ethics reference No. 14112), and which was prospectively registered with the Pan African Clinical Trial Registry on May 26, 2016 (http://www.pactr.org) (PACTR201706001651380).

## 3 RESULTS

Of the 615 women recruited at our center to the main ECHO Trial, 605 (98.4%) contributed data for at least one of the time‐points (3‐month or 12‐month follow up) in this ancillary study. At the 3‐month follow up, 188, 180, and 184 of these women had been randomized to receive DMPA‐IM, the IUD, and the LNG implant, respectively. Study interviews were conducted between September 2016 and March 2018 (Table 1).

**TABLE 1:** Participant follow up at three and twelve months

	**DMPA‐IM (n)**	**IUD (n)**	**LNG implant (n)**
Enrolled in ECHO trial	205	205	205
Cohort 1: 3‐month follow up			
Completed BDI	188	180	184
Completed ASEX	187	179	184
Lost to follow up	35	38	45
Additional data available	14	16	23
Cohort 2: 12‐month follow up			
Completed BDI	167	158	162
Completed ASEX	167	158	162
Completed WHO Well‐being Index	164	155	161
Completed PGI	166	156	158

Abbreviations: ASEX, Arizona Sexual Experiences Scale; BDI, Beck Depression Inventory; DMPA‐IM, depot medroxyprogesterone acetate; IUD, intrauterine device; LNG, levonorgestrel; PGI, Patient Global Impression.

Baseline characteristics of participants in the three study groups were similar, suggesting that the ECHO randomization process from which our study groups were derived was successful. Mean participant age of 25 years (standard deviation 4 years); approximately half of the women had a secondary school education (254/605, 42.0%); and few women were married (15/605, 2.5%) or employed (22/605, 3.6%).

There was no statistically significant difference between the study groups with respect to BDI or ASEX findings at 3 months (Table 2).

**TABLE 2:** Depression (BDI) and sexual function (ASEX) outcomes at 3 months after randomization^a^

Outcome	Allocated method			*p* value		
	DMPA‐IM (n = 188)	IUD (n = 180)	LNG implant (n = 184)	DMPA‐IM vs LNG implant	DMPA‐IM vs IUD	LNG implant vs IUD
Depression (BDI)						
Any depression (BDI ≥14)	63 (33.5%)	46 (25.6%)	51 (27.7%)	0.226	0.095	0.641
Moderate depression (BDI ≥20 and BDI ≤28)	20 (10.6%)	15 (8.3%)	16 (8.7%)	0.526	0.451	0.901
Severe depression (BDI ≥29)	12 (6.4%)	14 (7.8%)	18 (9.8%)	0.229	0.602	0.499
Moderate/Severe depression (BDI ≥20)	32 (17.0%)	29 (16.1%)	34 (18.5%)	0.713	0.814	0.551
Median BDI score	7.5 (1–16.5)	6 (0–14)	5 (0–16)	0.174	0.148	0.953
Sexual function (ASEX)^b^						
Sexual dysfunction^c^	40 (21.3%)	37 (20.6%)	41 (22.3%)	0.784	0.257	0.363
Mean ASEX score	12.4 ± 4.3	11.9 ± 4.4	12.3 ± 3.9	0.835	0.866	0.708

Abbreviations: ASEX, Arizona Sexual Experiences Scale; BDI, Beck Depression Inventory; DMPA‐IM, depot medroxyprogesterone acetate; IUD, intrauterine device; LNG, levonorgestrel.

^a^ Values are given as number (percentage), as median (interquartile range), or as mean ± standard deviation.

^b^ Two out of 552 participants did not contribute ASEX data.

^c^ Defined as an ASEX score of ≥19 or any one ASEX scale item with a score of >5 or any three items with a score of >4.^14^

At 12‐months, 167, 158, and 162 women contributed data for DMPA‐IM, IUD, and LNG implant groups, respectively. Among those that contributed data at the 3‐month follow up, loss to follow up at 12 months was similar across the three groups (434/552, 78.6%). Data were available for an additional 53 women at the 12‐month follow up (total *n* = 487). Significantly more women allocated to the IUD scored 20 or more (moderate or severe depression) on the BDI scale compared with those allocated to DMPA‐IM (28/158 [17.7%] vs. 16/167 [9.6%]; *p *= 0.032). In addition, median BDI scores were statistically significantly higher with LNG implants compared with DMPA‐IM (*p *= 0.036). A lower proportion of women in the DMPA‐IM group (24/167, 14.4%) experienced sexual dysfunction compared with the other two groups (32/158 [20.4%] and 33/162 [20.3%] for the IUD and LNG implant groups, respectively), but this did not reach statistical significance (Table 3).

**TABLE 3:** Depression (BDI) and sexual function (ASEX) outcomes at 12 months after randomization^a^

Outcome	Allocated method			*p* value		
	DMPA‐IM n = 167	IUD n = 158	LNG Implant n = 162	DMPA‐IM vs LNG Implant	DMPA‐IM vs IUD	LNG Implant vs IUD
Depression (BDI)						
Any depression (BDI ≥14)	29 (17.4%)	39 (24.7%)	33 (20.4%)	0.486	0.105	0.356
Moderate depression (BDI ≥20 and BDI ≤28)	9 (5.4%)	17 (10.8%)	12 (7.4%)	0.454	0.074	0.296
Severe depression (BDI ≥29)	7 (4.2%)	11 (7.0%)	9 (5.6%)	0.565	0.275	0.603
Moderate/Severe depression (BDI ≥20)	16 (9.6%)	28 (17.7%)	21 (13.0%)	0.332	0.032	0.237
Median BDI score	0 (0–9)	1 (0–13)	4 (0–11)	0.036	0.163	0.566
Sexual function (ASEX)						
Sexual dysfunction^b^	24 (14.4%)	32 (20.3%)	33 (20.4%)	0.151	0.161	0.979
Mean ASEX score	10.8 ± 4.0	11.1 ± 3.8	11.2 ± 4.0	0.365	0.475	0.839

Abbreviations: ASEX, Arizona Sexual Experiences Scale; BDI, Beck Depression Inventory; DMPA‐IM, depot medroxyprogesterone acetate; IUD, intrauterine device; LNG, levonorgestrel.

^a^ Values are given as number (percentage), as median (interquartile range), or as mean ± standard deviation.

^b^ Defined as an ASEX score of ≥19 or any one ASEX scale item with a score of >5 or any three items with a score of >4.^14^

For the WHO‐5 Well‐being Index, the median raw score of 19 was the same across the groups with slight, non‐significant differences in interquartile ranges of 16–22, 15–22 and 17–21 for the DMPA‐IM, LNG implant, and IUD groups, respectively (*p *= 0.913).

With the PGI scale, the majority of participants in each group felt “very much better”; however, significantly more participants using DMPA‐IM group felt “very much better” compared with those using the LNG implant and IUD (*p* = 0.003). Only one participant, in the LNG implant group, indicated that she felt “a little worse” (Table 4).

**TABLE 4:** Effects of DMPA‐IM, IUD, and LNG implant on Patient Global Impression^a^

Outcome	Allocated method			*P* value		
Patient Global Impression Scale	DMPA‐IM (n = 166)	IUD (n = 156)	LNG implant (n = 158)	DMPA‐IM vs LNG implant	DMPA‐IM vs IUD	LNG implant vs IUD
A little worse	0 (0.0%)	0 (0.0%)	1 (0.6%)	0.488	–	0.999
No change	1 (0.6%)	1 (0.6%)	3 (1.9%)	0.361	0.999	0.623
A little better	3 (1.8%)	14 (9.0%)	9 (5.7%)	0.064	0.004	0.265
Much better	16 (9.6%)	24 (15.4%)	26 (16.5%)	0.068	0.118	0.795
Very much better	146 (88.0%)	117 (75.0%)	119 (75.3%)	0.003	0.003	0.948

Abbreviations: DMPA‐IM, depot medroxyprogesterone acetate; IUD, intrauterine device; LNG, levonorgestrel.

^a^ Values are given as number (percentage).

At the end of the study, 466/487 women (95.7%) were still using contraception; however, 38/487 (7.8%) had changed from their study‐allocated method. Fewer women in the DMPA‐IM group changed their allocated method compared with the LNG implant and IUD groups (*p *= 0.002); most changes made were to DMPA‐IM from the IUD and LNG implant, 15/158 (9.5%) and 13/162 (8.0%) of women allocated to these methods, respectively (Table 5).

**TABLE 5:** Changes to allocated method^a^, ^b^

Outcome	DMPA‐IM (n = 167)	IUD (n = 162)	LNG implant (n = 158)	*P* value
Changed method	8 (4.8%)	25 (15.8%)	26 (16.1%)	0.002
New method				
Changed to DMPA‐IM	–	15 (9.5%)	13 (8.0%)	0.642
Changed to IUD	0 (0.0%)	–	0 (0.0%)	–
Changed to LNG implant	2 (1.2%)	0 (0.0%)	–	0.168
Changed to other method	1 (0.6%)	2 (1.3%)	5 (3.1%)	0.187
Not on a method	5 (3.0%)	8 (5.1%)	8 (4.9%)	0.585

Abbreviations: DMPA‐IM, depot medroxyprogesterone acetate; IUD, intrauterine device; LNG, levonorgestrel.

^a^ Values are given as number (percentage).

^b^ Despite these changes, all analyses were by intention to treat.

## 4 DISCUSSION

This prospective study conducted alongside the ECHO Trial at one of the ECHO Trial sites (the Effective Care Research Unit) in South Africa at 3 and 12 months after randomization found that at 12 months women using DMPA‐IM were at lower risk of depressive symptoms than women using the IUD or the LNG implant and were more likely to feel “very much better” on the PGI scale. No significant differences in the risk of sexual dysfunction or sexual function scores were found.

Before conducting the study, we hypothesized that depressive symptoms would be higher with the progestin‐only contraceptives based on previous studies that have found depression to be increased among users of postnatal injectable hormonal contraceptives.^5^, ^6^ This is the first prospective study associated with a randomized trial where follow up with respect to these outcomes has been extended to 12 months after contraception initiation, and in a non‐postnatal cohort. Rates of any depression in this ancillary study were lower than that seen in a previous study in our setting ^9^ and may be due to the difference in the population group (postnatal in the previous study) and the duration of follow up (longer in the current study). The rate of any depression at 3 months after contraception initiation was 33.5% among DMPA‐IM users compared with 17.4% at the 12‐month follow up, suggesting that the risk of depression may decrease over time, particularly for DMPA‐IM users.

The ECHO trial findings show that women using the IUD were more likely to experience menorrhagia, pelvic pain, dysfunctional uterine bleeding, and dysmenorrhea than those using DMPA‐IM.^3^ Dysfunctional uterine bleeding was also more common with the LNG implant than with DMPA‐IM in the ECHO trial. Therefore, it is plausible that the higher rates of depressive symptoms with the IUD and the LNG implant at 12 months may be related to these troublesome menstrual disturbances.

Although this ancillary study recruited women after randomization, it included 90% of the originally randomized participants, and the proportion of ECHO‐randomized participants in each intervention group was similar at both time‐points evaluated. In addition, because of the alignment of this ancillary study with the stringent follow up of the associated rigorous RCT, 12‐month follow up was high. These study strengths mean that meaningful bias due to selective participation is unlikely. Another strength is that we performed intention‐to‐treat analysis; therefore, the effect of more women switching to DMPA‐IM than to the other methods would have had the effect of under‐estimating effects of DMPA‐IM rather than over‐estimating them.

A limitation of the study is that we did not collect data on mood and sexual functioning at baseline; neither did we record the use of anti‐depressant medication or counseling; however, such health interventions are rare among women in our setting. Another limitation is that we did not evaluate previous contraceptive use among women in our study, which may have influenced the potential for women to switch methods or to discontinue a given method. As suggested by greater voluntary switching to DMPA‐IM than to other methods, DMPA‐IM is a very popular contraceptive method among contraceptive users in our setting. This may have influenced the findings of our study in that women consenting to participate in the trial and allocated to DMPA‐IM may have been relatively happier with their allocated method than those allocated to the other two methods. Outside the ECHO trial context, contraceptive use is entirely based on personal preference; hence, our findings may not be applicable to the usual context in which women choose their contraceptive method. Nevertheless, the findings on mood should go some way to reassure DMPA‐IM users that this method probably does not cause a low mood compared with other contraceptive agents and is usually associated with a reasonable sense of well‐being. Similarly, the findings of no difference in libido should be reassuring to users of all three contraceptive methods evaluated. The higher rate of low mood among IUD users found in this study, in addition to the known menstrual adverse effects, may have contributed to the higher discontinuation rates seen among IUD users compared with DMPA‐IM users in the ECHO Trial (9% versus 4%),^3^ and confirms the importance of counseling prospective users about these adverse effects and managing them effectively when they arise.

In conclusion, major differences in sexual functioning between women receiving DMPA‐IM, the IUD, and the LNG implant are unlikely. DMPA‐IM use in South Africa is probably associated with a lower risk of depression at 12 months after contraceptive initiation; however, evidence from other settings is needed.

## DATA AVAILABILITY STATEMENT

We support data sharing and such requests can be emailed to Mandisa Singata‐Madliki (mandisa.singata@gmail.com).

## CONFLICTS OF INTEREST

The authors have no conflicts of interest.

## AUTHOR CONTRIBUTIONS

MS‐M conceived the study and contributed to its conduct and to writing of the manuscript. JH contributed to study design, interpretation of data and writing of the manuscript. TL contributed to study design and wrote the first draft of the manuscript. YB contributed to the study design, data analysis and writing of the manuscript. FC‐Ld’H contributed to study design, and reviewed and advised on the draft manuscript. All authors approved of and contributed to the final manuscript.
